# Genetic Algorithm-Assisted Multilayer SPR Refractive-Index Sensor with FASnI_3_ Perovskite and Black Phosphorus: A Theoretical Study

**DOI:** 10.3390/s26175669

**Published:** 2026-09-07

**Authors:** Chaoye Yao, Jiquan Lan, Haoyuan Cai

**Affiliations:** 1 College of Ocean Information Engineering, Jimei University, Xiamen 361021, China; chaoyeyao2026@163.com; 2 Fujian Provincial Key Laboratory of Oceanic Information Perception and Intelligent Processing, School of Ocean Information Engineering, Jimei University, Xiamen 361021, China; 3 Fujian Branch, China Construction Eighth Engineering Bureau Co., Ltd., Xiamen 361008, China

**Keywords:** surface plasmon resonance, refractive-index sensor, genetic algorithm, composite sensitivity figure, FASnI_3_

## Abstract

Surface plasmon resonance (SPR) sensors translate refractive-index (RI) changes near an interface into measurable angular shifts, but a large shift is useful only when the resonance remains sufficiently narrow and deep. Conventional single-metal SPR structures typically force a trade-off in which sensitivity gains come at the cost of broader resonances or shallower reflectance dips. Here, a BAK1/Cu/Al/BaTiO_3_/FASnI_3_/BP multilayer SPR refractive-index sensor is proposed and optimized using the transfer matrix method (TMM) coupled with a genetic algorithm (GA). The Cu/Al bimetallic region provides a plasmonic metal core, BaTiO_3_ and FASnI_3_ progressively enhance the evanescent field, and black phosphorus (BP) forms the analyte-facing sensing interface. To avoid sensitivity-only optimization, the GA uses a composite sensitivity figure (CSF) that integrates angular sensitivity, resonance dip depth, and full width at half maximum as the fitness function. At an analyte refractive index (RI) of 1.355, the sensor reaches a maximum sensitivity of 510.11°/RIU and a CSF of 76.39 RIU^−1^. These results establish the GA-CSF framework as a generalizable route to the balanced design of multilayer SPR refractive-index sensors and provide a computationally guided starting point for experimental implementation.

## 1. Introduction

Surface plasmon resonance (SPR) is a label-free optical technique that converts refractive-index (RI) variations near a sensing interface into measurable resonance-angle shifts [1,2]. Because interfacial refractive-index (RI) changes occur in chemical, environmental, food-quality, and biological analyses, SPR architectures are applicable to a broad range of analytical measurements. In angular-interrogation SPR sensors, however, a large resonance-angle shift is useful only when the resonance remains sufficiently narrow and deep to be resolved accurately. Accordingly, recent SPR studies increasingly evaluate linewidth, dip depth, detection accuracy, and limit of detection alongside sensitivity [3,4].

Achieving this balance with a conventional single-metal stack remains challenging. The coupling prism determines momentum matching to the surface plasmon mode, while the metal layer introduces a stability–loss tradeoff: Gold (Au) is chemically stable but can broaden the resonance, Silver (Ag) has a strong plasmonic response but oxidizes readily, and Copper (Cu) offers strong plasmonic activity and cost-effectiveness but requires protection in aqueous environments [5,6,7]. Aluminum, by contrast, possesses a high electron density and a substantially more negative real permittivity than conventional noble metals, enabling efficient conversion of optical energy into surface plasmons [8,9]. To overcome the limitations of monometallic configurations, bimetallic and multimaterial architectures have attracted considerable attention as a route to simultaneously improve resonance sharpness, chemical durability, and field confinement [10,11,12].

Beyond the metal core, the evanescent field must be enhanced so that minute refractive-index changes at the sensing interface yield a measurable resonance shift. Barium titanate (BaTiO_3_), a perovskite-structured oxide with a high dielectric constant, has been shown to produce larger resonance-angle shifts and lower reflection intensity than alternative perovskite materials [13,14,15]. With a refractive index of 2.4042 at 633 nm, BaTiO_3_ enhances the surface-bound field and protects the underlying metal film from oxidation [16,17]. Additional field amplification is provided by a halide perovskite layer. Formamidinium tin iodide (FASnI_3_), a lead-free three-dimensional halide perovskite with a direct transition, a long carrier diffusion length of 10^2^–10^5^ nm, high carrier mobility (10–10^2^ cm^2^V^−1^S^−1^), and a large absorption coefficient (>10^4^ cm^−1^), deepens the resonance dip and sharpens the spectral profile beyond what the dielectric layer alone can achieve [18,19,20,21]. Incorporating FASnI_3_ promotes deeper resonance dips and sharper spectral features compared with metal-only configurations [18,21].

To further enhance interfacial RI sensitivity, black phosphorus (BP) is introduced as the topmost sensing layer [22]. Compared with graphene and MoS_2_, BP exhibits substantially stronger optical absorption, rendering it more responsive to ambient RI perturbations. Unlike graphene, BP is a direct-bandgap material (tunable from ~0.3 eV in bulk to ~2.0 eV in monolayer) with remarkable carrier mobility and unique in-plane anisotropic structure, enabling deeper reflection dips and enhanced signal contrast at the sensing interface [23].

While each of these layers contributes a distinct performance benefit, their effects are coupled in the assembled stack rather than independent. A thickness change that strengthens the field may simultaneously broaden the resonance, reduce the dip depth, or move the optimum point outside the desired angular range. The number of BP layers also introduces a discrete design variable. Manual scans are therefore inefficient and can favor locally optimal but practically unbalanced structures.

In this work, the transfer matrix method (TMM) is coupled with a genetic algorithm (GA) so that the multilayer sensing structure stack can be evaluated as one optical system. The fitness function is the composite sensitivity figure (CSF), which integrates angular sensitivity, resonance depth, and full width at half maximum, rewarding high angular sensitivity only when it is accompanied by sufficient resonance depth and acceptable linewidth. Under this framework, the proposed BAK1/Cu/Al/BaTiO_3_/FASnI_3_/BP sensor is evaluated from structural, optical, robustness, and application perspectives. The analysis compares candidate prism materials, optimizes multilayer thicknesses, benchmarks the optimized device against reported SPR structures and evaluates fabrication tolerance.

## 2. Materials and Methods

### 2.1. Sensor Architecture and Functional Design

**Figure 1:**
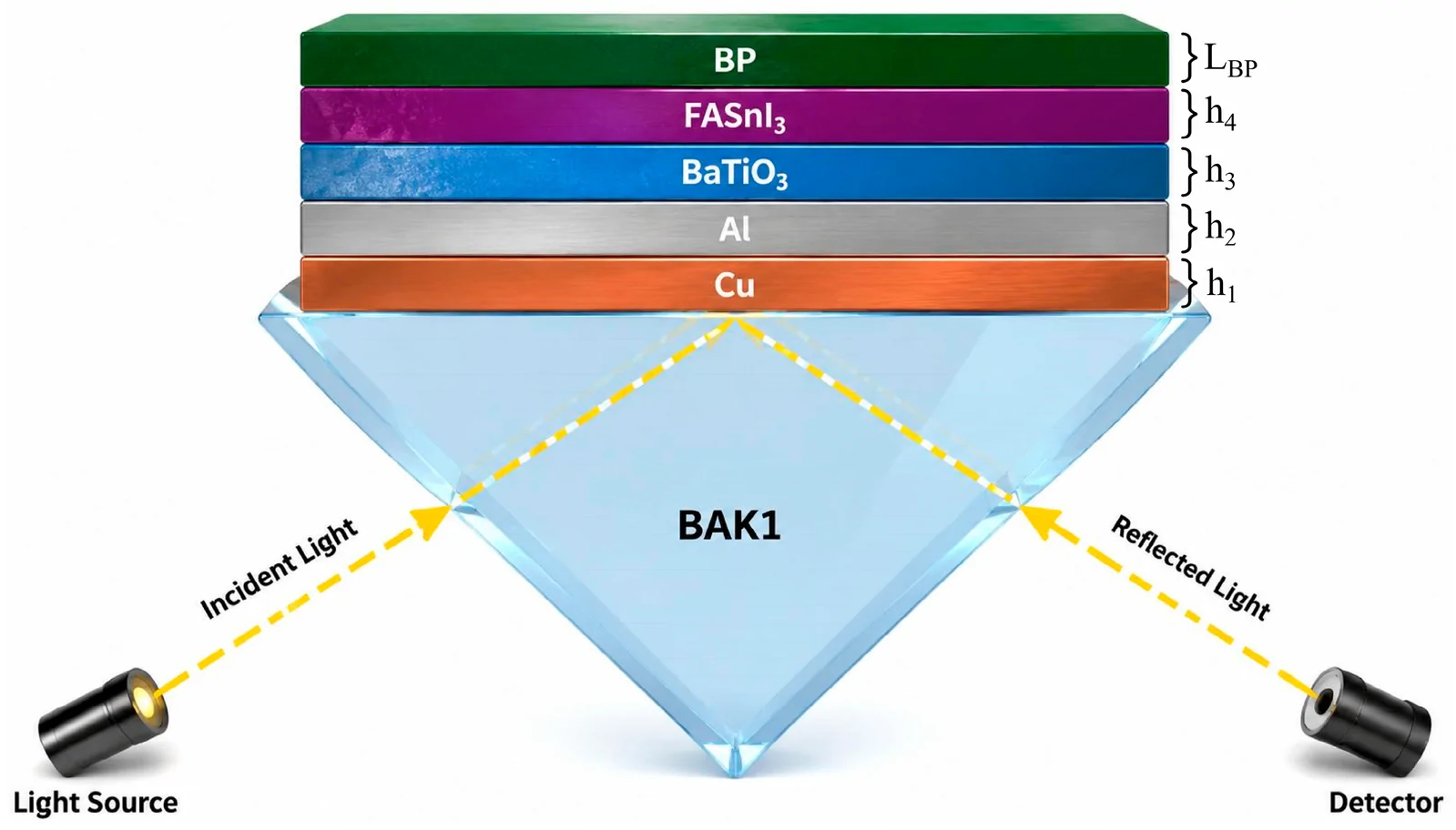
Schematic illustration of the proposed BAK1/Cu/Al/BaTiO_3_/FASnI_3_/BP SPR refractive-index sensor in the Kretschmann configuration.

The proposed refractive-index sensor adopts the Kretschmann arrangement, depicted in Figure 1. A BAK1 prism is used as the final coupling medium after comparison with F2, SF2, BAF10, and SF5. Adjacent to the prism, the first functional layer is a Cu film of thickness h_1_ that serves as the primary plasmonic transducer. An Al film of thickness h_2_ is placed on the Cu layer as a protective and plasmonic capping film. A BaTiO_3_ dielectric layer of thickness h_3_ is then introduced to enhance the evanescent field, followed by a FASnI_3_ halide perovskite layer of thickness h_4_. The outermost sensing interface consists of L_BP_ layers of BP deposited on FASnI_3_. The material optical constants used in the calculations are summarized in Table 1.

### 2.2. Performance Metrics

The angular reflectance of the planar multilayer stack is calculated using TMM. The resonance angle is the angle of minimum reflectance. The angular sensitivity S is quantified by the change in resonance angle per unit refractive-index variation. A higher S value indicates that a smaller change in sensing-medium RI produces a larger angular response. The full width at half maximum (FWHM) describes the spectral width of the reflectance dip. A smaller FWHM reflects a sharper resonance profile and generally improves the ability to identify small angle shifts. The dip reflection depth (DRD) describes the contrast of the SPR minimum and is calculated from the normalized reflectance minimum as DRD = 1 − Rmin, where Rmin is the minimum normalized reflectance at the resonance angle.

For the uniformly stacked multilayer architecture depicted in Figure 1, the TMM is employed to model and analyze the *N*-layer structure [27]. The material layers are assumed to be stacked along the *z* axis, and the properties of each material layer are defined by its dielectric constant and thickness. Based on Maxwell’s equations, the *N*-layer structure can be described by the following matrix formalism, from which the reflectance of the multilayer stack is calculated.

According to TMM, the tangential components of the electric field (*E*_1_) and the magnetic field (*H*_1_) at the boundary of the first layer and those (*E_N_*_−1_, *H_N_*_−1_) at the boundary of the last layer are related by the following matrix equation:

$$\left[\begin{array}{c} E_{1}\\ H_{1} \end{array}\right]=M\left[\begin{array}{c} E_{N-1}\\ H_{N-1} \end{array}\right]$$

where *E*_1_ and *H*_1_ denote the tangential components of the electric and magnetic fields at the boundary of the first layer, respectively, and *E_N_*_−1_ and *H_N_*_−1_ denote those at the boundary of the last layer. *M* is the characteristic transfer matrix of the N-layer structure, which is given by:

$$M=\prod_{k=2}^{N-1}M_{k}=\left[\begin{array}{cc} M_{11} & M_{12}\\ M_{21} & M_{22} \end{array}\right]$$

with

$$M_{k}=\left[\begin{array}{cc} \cos\beta_{k} & (-i\sin\beta_{k})/q_{k}\\ -iq_{k}\sin\beta_{k} & \cos\beta_{k} \end{array}\right]$$

where *β*_k_ and *q_k_* represent the phase thickness and the optical admittance of the *k*-th layer for p-polarized incidence, respectively, which are given by

$$\beta_{k}=\frac{2\pi d_{k}}{\lambda }{\left(\varepsilon_{k}-n_{1}^{2}\sin\theta_{1}\right)}^{1/2}$$

$$q_{k}=\frac{{\left(\varepsilon_{k}-n_{1}^{2}\sin\theta_{1}\right)}^{1/2}}{\varepsilon_{k}}$$

where *d*_k_ and *ε*_k_ denote the thickness and dielectric constant of the *k*-th layer, respectively, *n*_1_ is the refractive index of the incident medium (prism), *θ*_1_ is the incident angle, and *λ* is the wavelength of the incident light. The amplitude reflection coefficient of the p-polarized incident wave is then expressed as:

$$r_{p}=\frac{\left(M_{11}+M_{12}q_{N}\right)q_{1}-\left(M_{21}+M_{22}q_{N}\right)}{\left(M_{11}+M_{12}q_{N}\right)q_{1}+\left(M_{21}+M_{22}q_{N}\right)}$$

Since only p-polarized light can excite surface plasmon waves (SPWs), the reflectance of the SPR multilayer configuration is finally given by

$$R_{p}={\left|r_{p}\right|}^{2}$$

The resonance angle (θ_res_) was extracted from the angular reflectance spectrum of the multilayer stack. Reflectance was computed as a function of the incident angle (θ_1_) over the range 60–89° with a step size of 0.001°. The resonance angle was identified as the angle of minimum reflectance corresponding to the sharp SPR dip, and the FWHM was determined by linear interpolation between the half-maximum crossing points on either side of the dip.

Because these quantities are coupled, the composite sensitivity figure is used as the main optimization metric: CSF = S × DRD/FWHM. This definition integrates angular sensitivity, resonance contrast, and linewidth into a single ranking criterion.

### 2.3. GA-Assisted Optimization Framework

The optimization variable is x = (h_1_, h_2_, h_3_, h_4_, L_BP_), where h_1_, h_2_, h_3_, and h_4_ are the thicknesses of Cu, Al, BaTiO_3_, and FASnI_3_, respectively, and L_BP_ denotes the layer count of BP. The GA starts from a randomly generated population of 400 individuals constrained by the parameter ranges in Table 2. The detailed GA parameters are listed in Table 3. The algorithm evaluates each candidate design via TMM-generated angular reflectance spectra, extracts S, FWHM, and DRD, and ranks individuals by their CSF values. Selection, crossover, and mutation then update the population iteratively until the best CSF converges. In this way, the algorithm simultaneously searches for the continuous metal, dielectric, and perovskite thicknesses and the discrete BP layer number.

## 3. Results

### 3.1. Prism Selection and Comparative Analysis

**Figure 2:**
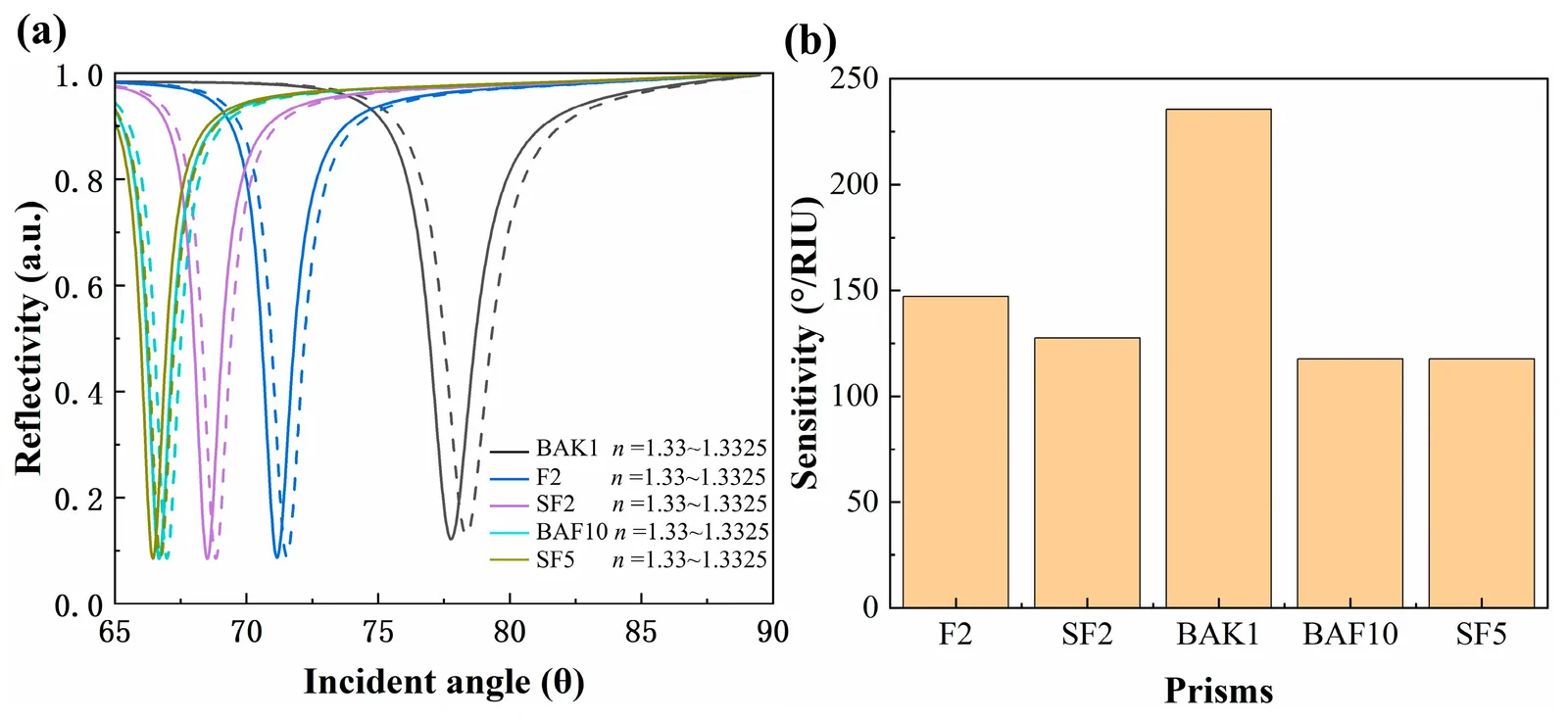
Comparative analysis of five prism materials for the complete Cu/Al/BaTiO_3_/FASnI_3_/BP multilayer architecture. (**a**) Simulated angular reflectance spectra at analyte RI 1.33 (solid) and 1.3325 (dashed). (**b**) Corresponding angular sensitivity comparison.

Before GA-based layer optimization, five candidate prism materials were screened under the same multilayer architecture: F2, SF2, BAK1, BAF10, and SF5. Because the prism primarily controls momentum matching and the accessible resonance-angle window, this screening step focused on angular displacement and spectral readability. Figure 2a presents the simulated reflectance spectra at analyte RI values of 1.3300 (solid curves) and 1.3325 (dashed curves). Marked variations were observed in the SPR responses across the candidate prisms. As the prism refractive index changed from BAK1 to F2, SF2, BAF10, and SF5, the resonant coupling angle shifted from approximately 77.8° to 71.2°, 68.5°, 66.7°, and 66.4°, respectively. BAK1 produced the largest resonance-angle displacement within the investigated refractive-index window of Δn = 0.0025, indicating the highest angular sensitivity at the prism-selection stage. Figure 2b quantifies the angular sensitivity obtained from this preliminary prism comparison. BAK1 achieved the highest value of 235.44°/RIU, exceeding F2 (147.15°/RIU), SF2 (127.53°/RIU), BAF10 (117.72°/RIU), and SF5 (117.72°/RIU). This result indicates that BAK1 provides the most favorable momentum matching for the proposed multilayer architecture before thickness optimization. The prism choice is therefore based on the pre-optimization sensitivity and spectral response.

### 3.2. GA-Based Structural Optimization

**Figure 3:**
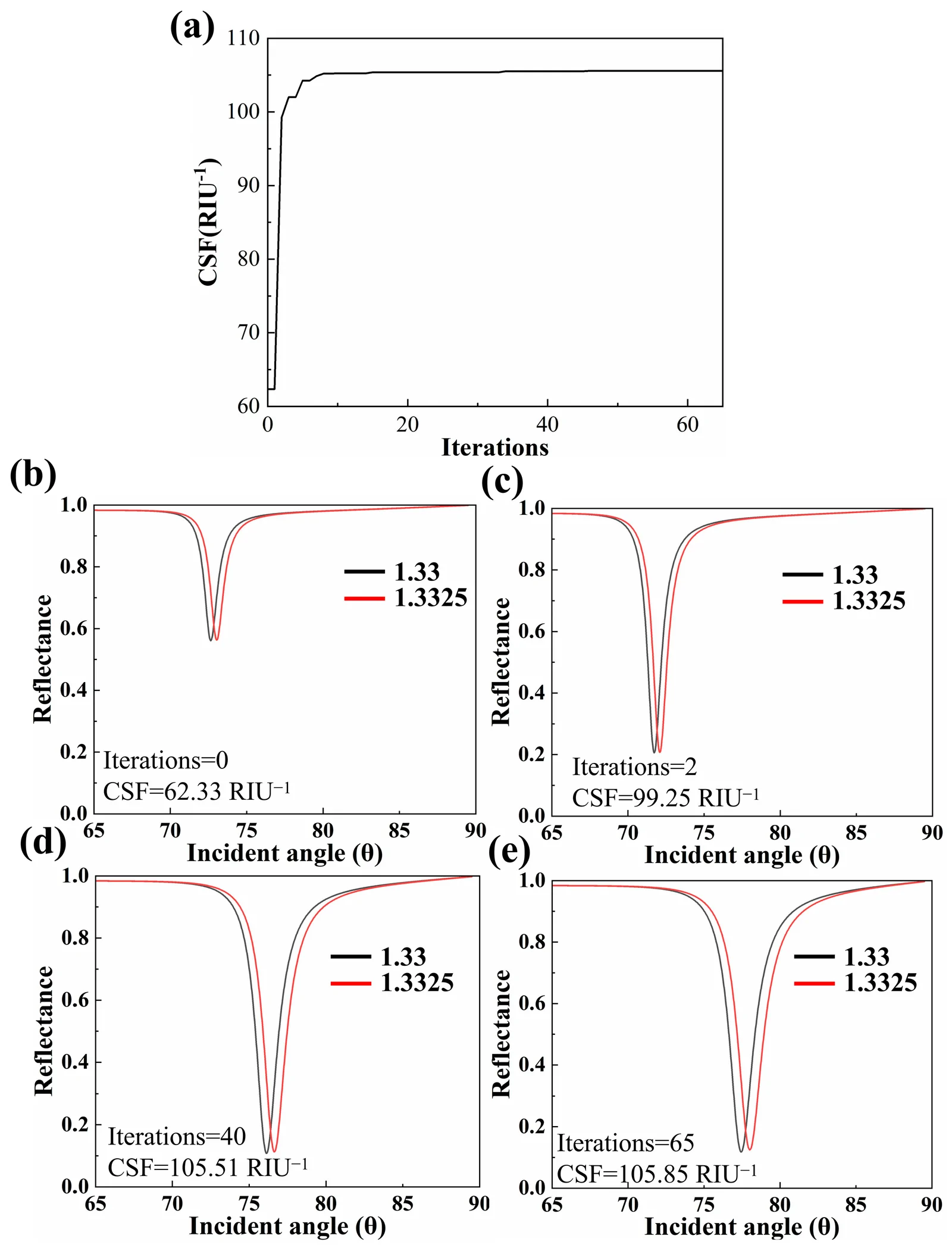
Genetic algorithm optimization of the BAK1/Cu/Al/BaTiO_3_/FASnI_3_/BP SPR sensor. (**a**) CSF convergence during evolution. (**b**–**e**) Angular reflectance spectra at iterations 0, 2, 40, and 65.

Figure 3a presents the convergence curve of CSF during the GA evolution. In the initial stage, the CSF of the best individual in the population rises sharply from 62.34 RIU^−1^ at Iterations = 0, demonstrating strong global search capability. The growth rate then slows, reaching 105.51 RIU^−1^ at iteration 40 and converging to 105.85 RIU^−1^ at iteration 65, with an overall optimization gain of approximately 70%. The resulting optimal geometry is h_1_ = 58.15 nm for Cu, h_2_ = 2.2 nm for Al, h_3_ = 1.8 nm for BaTiO_3_, h_4_ = 12.27 nm for FASnI_3_, and L_BP_ = 1.

Figure 3b–e shows the angular reflectance curves at different evolutionary generations, where the black trace represents *n* = 1.3300, and the red trace corresponds to *n* = 1.3325. At iteration 0 (Figure 3b), the resonance dip is broad and shallow, with minimal angular separation between the two traces. By iteration 2 (Figure 3c), the dip has deepened and narrowed considerably, yet the angular shift remains modest. At iteration 40 (Figure 3d), the resonance becomes noticeably sharper, and the angular separation between the two RI conditions increases markedly. By iteration 65 (Figure 3e), the dip is at its sharpest and deepest, with the largest angular spacing near resonance. The optimization therefore improves not only angular sensitivity but also the sharpness and depth of the SPR response.

### 3.3. Performance Characterization and Comparative Analysis of Sensor Structures

A layer-by-layer comparison was performed to identify the contribution of each functional layer. Three structures were analyzed: the baseline without FASnI_3_ and BP, the intermediate with FASnI_3_, and the complete sensor with all layers. Simulations used GA-optimized parameters and TMM calculations across an analyte RI of 1.3300–1.3325.

**Figure 4:**
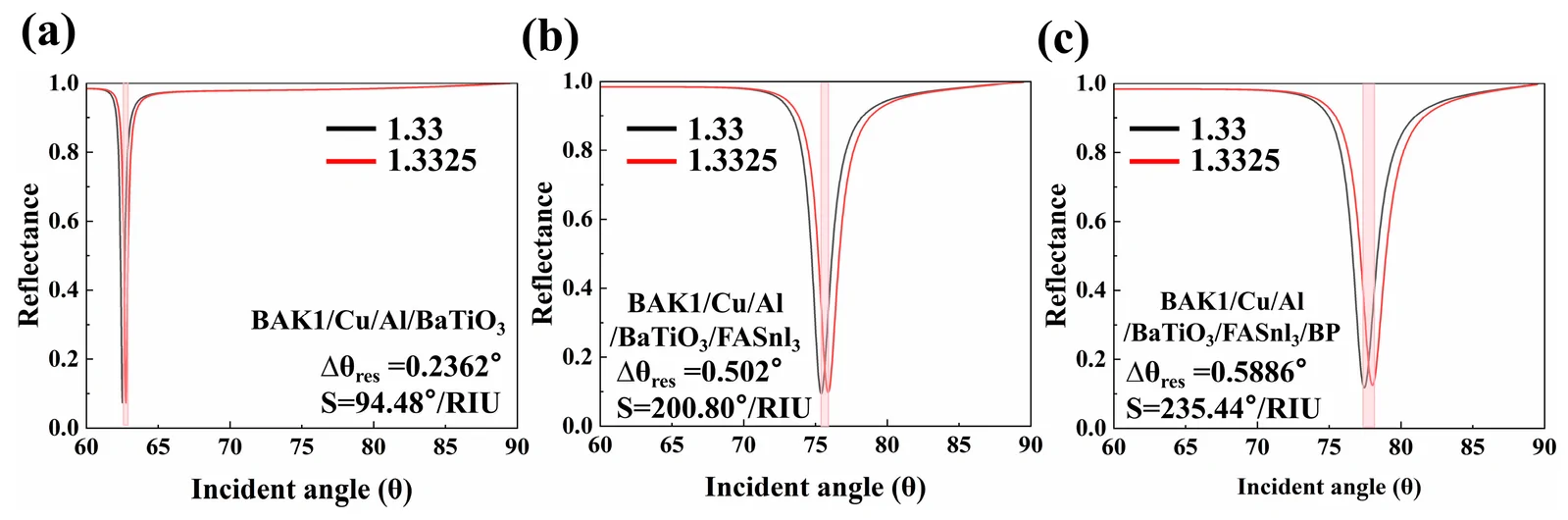
Simulated angular reflectance spectra for different sensor architectures as analyte RI varies from 1.3300 to 1.3325: (**a**) BAK1/Cu/Al/BaTiO_3_, (**b**) BAK1/Cu/Al/BaTiO_3_/FASnI_3_, and (**c**) BAK1/Cu/Al/BaTiO_3_/FASnI_3_/BP.

As shown in Figure 4a, the baseline stack structure exhibits a resonance-angle shift of 0.2362°, corresponding to an angular sensitivity of 94.48°/RIU. In this configuration, the Cu layer serves as the primary plasmonic transducer, the Al capping layer helps protect the Cu surface while supporting plasmon excitation, and the BaTiO_3_ overlayer projects the localized electromagnetic field toward the analyte medium. After integration of the FASnI_3_ perovskite layer (Figure 4b), the same RI increment produces a resonance shift of 0.502°, increasing the sensitivity to 200.80°/RIU, a 2.13-fold amplification. The complete heterostructure with monolayer BP as the terminal sensing element (Figure 4c) achieves a further resonance shift of 0.5886°, corresponding to 235.44°/RIU. This stepwise increase from 94.48 to 200.80 and then to 235.44°/RIU supports the cumulative field-amplification role of BaTiO_3_, FASnI_3_, and BP, with the high-permittivity dielectric, halide perovskite, and two-dimensional BP layers progressively concentrating the evanescent field in the analyte-proximal region.

**Figure 5:**
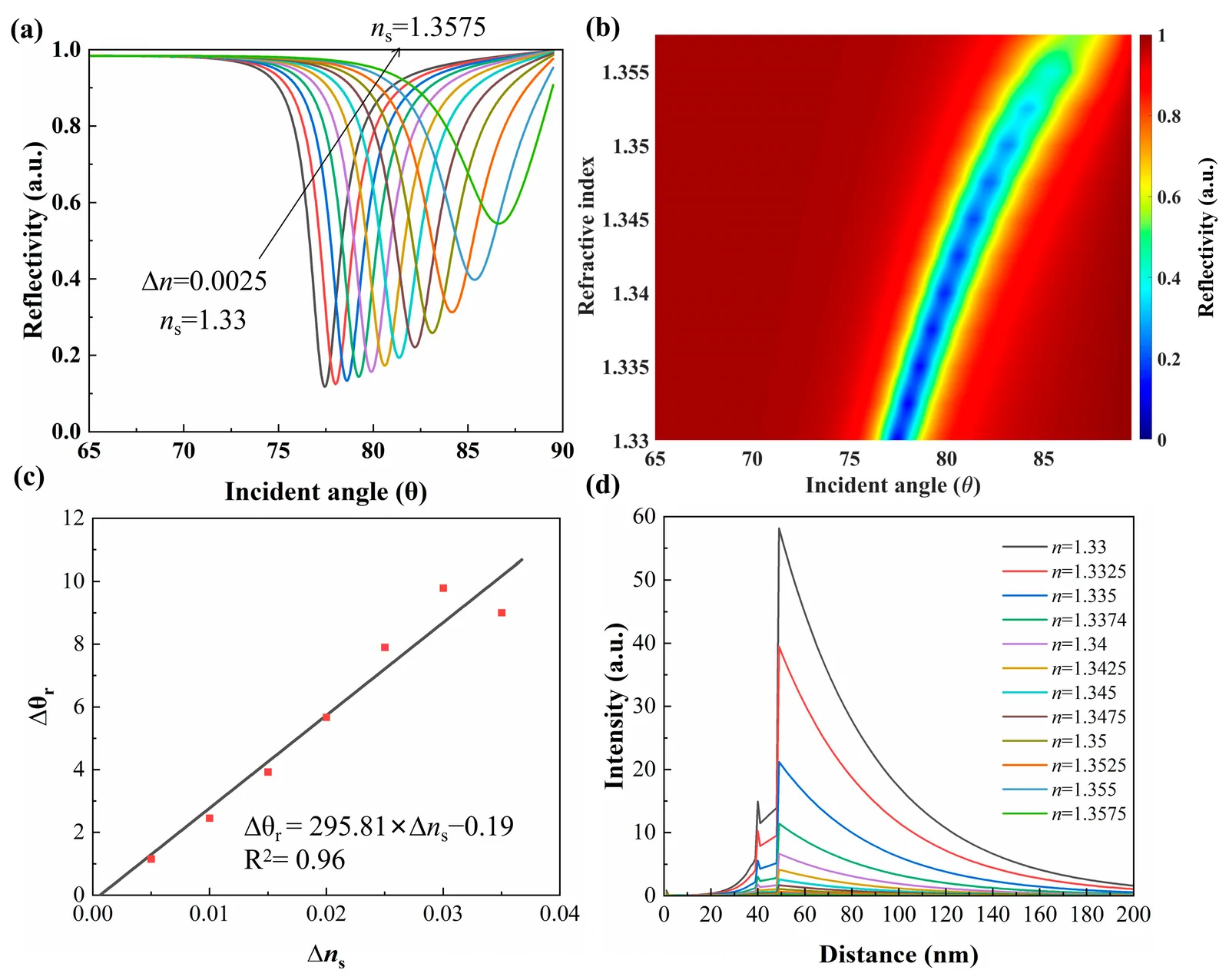
Comprehensive performance characterization of the optimized BAK1/Cu/Al/BaTiO_3_/FASnI_3_/BP sensor: (**a**) angular reflectance spectra for analyte RI = 1.3300–1.3575, (**b**) reflectance contour map, (**c**) linear regression of resonance-angle shift versus RI change, and (**d**) electric-field intensity distribution.

To validate the full operational capabilities of the optimized BAK1/Cu/Al/BaTiO_3_/FASnI_3_/BP sensor, a comprehensive characterization was conducted across the analyte refractive index range of 1.330 to 1.3575. Figure 5a presents the angular reflectance spectra as the analyte RI increases in 0.0025 increments. Each curve exhibits a distinct resonance dip, and the entire spectrum monotonically shifts toward larger incident angles with increasing sensing medium refractive index, reflecting the increased propagation constant of the surface plasmon wave. The contour map in Figure 5b plots reflectance as a function of incident angle and RI, revealing a highly regular resonance trajectory. Linear regression performed on the resonance locations (Figure 5c) yields an average sensitivity of 295.81°/RIU with R^2^ = 0.96, confirming that the sensor responds quantitatively across its full operational range. Figure 5d displays the electric-field intensity distribution at each resonance angle. The field remains tightly confined within the Cu/Al bimetallic region and changes noticeably near the sensing interface as the analyte RI varies, confirming the high RI responsivity of the proposed architecture.

Table 4 presents the simulated performance of the optimized sensor as the analyte refractive index increases from 1.330 to 1.3575. The angular sensitivity rises monotonically with RI, reaching a maximum of 510.11°/RIU at 1.355 before dropping to 245.25°/RIU at 1.3575. Meanwhile, the resonance peak broadens gradually, with FWHM increasing from 1.96° to 4.59°, and the dip reflection depth decreases steadily, with DRD falling from 0.882 to 0.455, indicating shallower resonance profiles at higher refractive indices. The CSF decreases overall, but stays above 76 throughout the 1.330–1.355 range.

**Figure 6:**
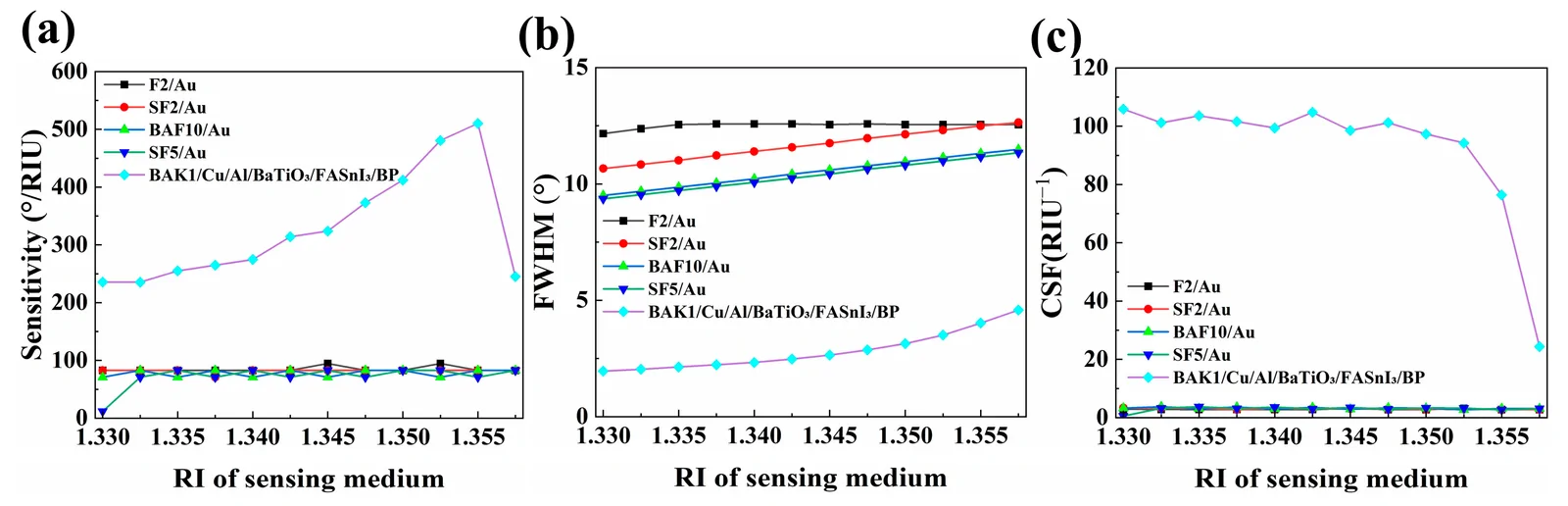
Comparative performance of the proposed BAK1/Cu/Al/BaTiO_3_/FASnI_3_/BP sensor and conventional F2/Au, SF2/Au, BAF10/Au, and SF5/Au benchmarks across analyte RI = 1.3300–1.3575: (**a**) angular sensitivity, (**b**) FWHM, and (**c**) CSF.

Figure 6 compares the optimized sensor with conventional F2/Au, SF2/Au, BAF10/Au, and SF5/Au benchmarks. Each benchmark adopts the conventional Kretschmann configuration, consisting solely of the coupling prism coated with a single Au film, and was evaluated under the same simulation conditions as the proposed sensor. At RI = 1.355, the optimized heterostructure achieves an angular sensitivity of 510.11°/RIU (Figure 6a), representing a 6.17-fold improvement over the conventional benchmarks, whose sensitivities remain approximately 82.68°/RIU. Figure 6b shows that the proposed sensor attains an FWHM of 4.02°, substantially narrower than the conventional structures (11.16° for SF5/Au, 11.31° for BAF10/Au, 12.49° for SF2/Au, and 12.55° for F2/Au). As shown in Figure 6c, the optimized architecture achieves a CSF of 76.39 RIU^−1^, outperforming the conventional benchmarks by factors ranging from 24.4 for BAF10/Au (3.13 RIU^−1^) to 28.1 for SF5/Au (2.72 RIU^−1^). The sensitivity peak at this refractive index arises from the constructive interplay between FASnI_3_-enhanced photon harvesting and BP-mediated near-field confinement. These results demonstrate that the GA-optimized multilayer architecture delivers peak performance in the biologically relevant high-RI regime.

### 3.4. Fabrication Tolerance and Robustness Analysis

**Figure 7:**
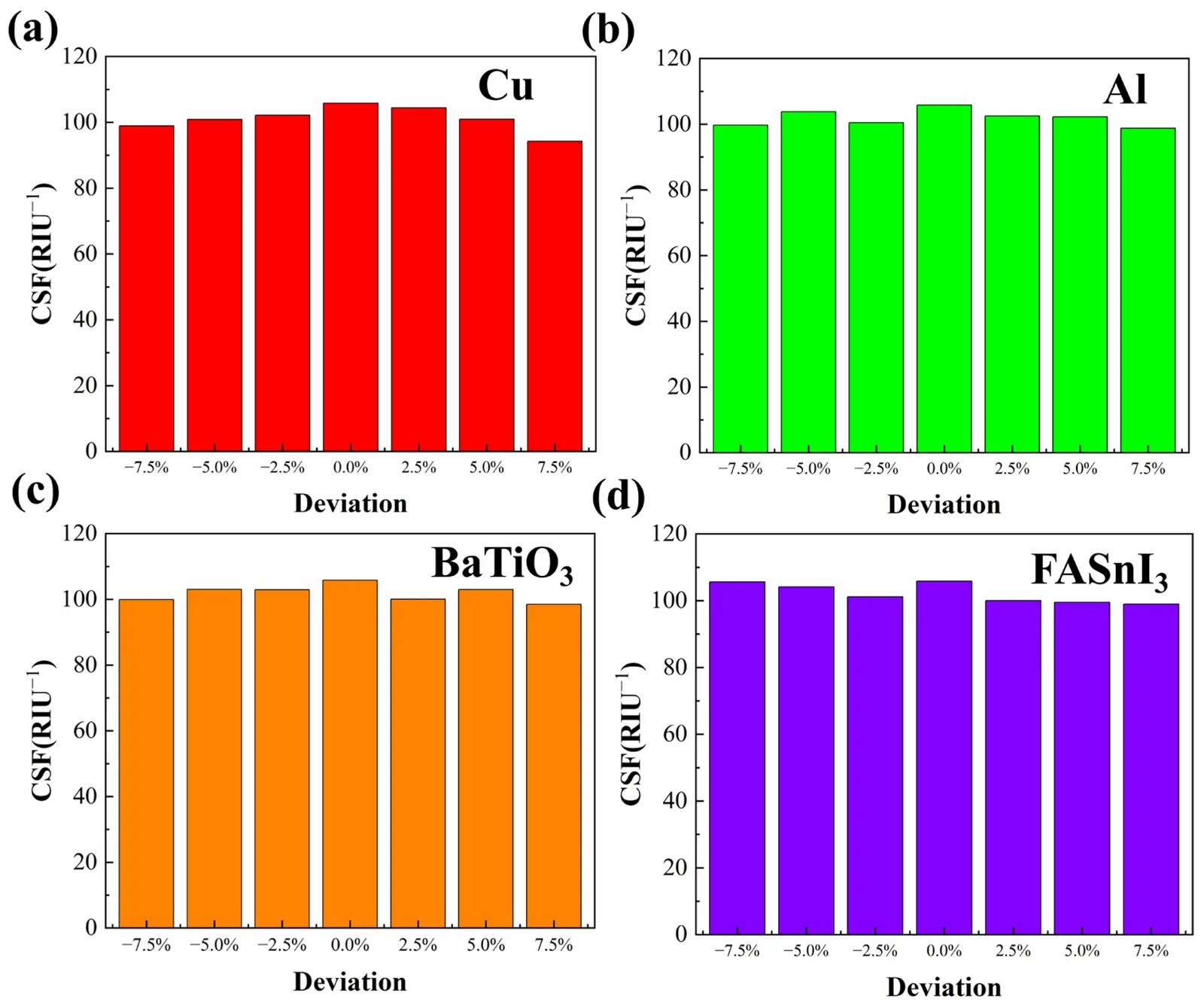
Fabrication tolerance analysis showing CSF variation with thickness deviation for (**a**) Cu, (**b**) Al, (**c**) BaTiO_3_, and (**d**) FASnI_3_ layers.

Fabrication tolerance was evaluated by introducing thickness deviations of −7.5% to +7.5% for the Cu, Al, BaTiO_3_, and FASnI_3_ layers while monitoring the CSF at an analyte RI of 1.330. As shown in Figure 7, the CSF varies nonlinearly with thickness deviation for all four layers, and the nominal GA-optimized design (0% deviation) consistently attains the highest CSF (105.85 RIU^−1^) in every sweep. FASnI_3_ exhibits a generally decreasing CSF trend with increasing thickness, from 105.66 RIU^−1^ at −7.5% (less than 0.2% below the nominal value) to 98.97 RIU^−1^ at +7.5%. The Cu layer shows the strongest overall variation: overdeposition reduces the CSF rapidly, reaching the lowest value of all sweeps (94.2 RIU^−1^) at +7.5%, whereas at +2.5% the CSF still reaches 104.4 RIU^−1^. By contrast, the Al and BaTiO_3_ layers display flatter responses, with their minimum CSF values of 98.78 and 98.53 RIU^−1^, respectively, occurring near the edges of the swept range. Even under the most unfavorable perturbation, the CSF remains above 94.2 RIU^−1^, confirming that the GA-optimized architecture maintains a substantial performance margin under realistic fabrication uncertainty.

Table 5 benchmarks the proposed sensor against recently reported SPR structures. Because reported performance metrics vary among studies, angular sensitivity is used as the primary comparison parameter, while CSF is included when available. The proposed BAK1/Cu/Al/BaTiO_3_/FASnI_3_/BP sensor maintains a high angular sensitivity among the listed structures and also provides a strong CSF value, indicating that the sensitivity enhancement is accompanied by favorable linewidth and dip-depth characteristics rather than arising from sensitivity alone.

### 3.5. Fabrication Feasibility and Process Roadmap

**Figure 8:**
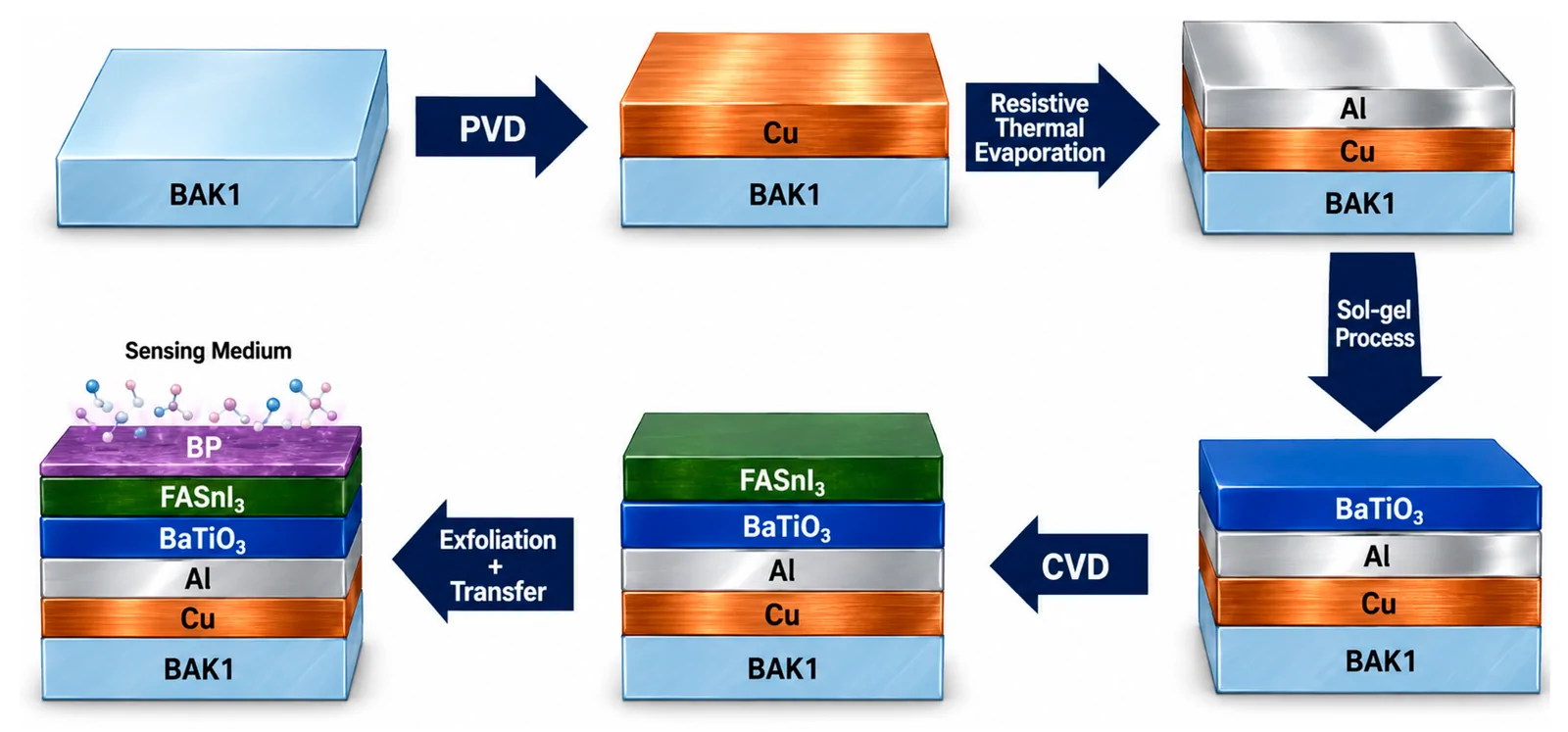
Schematic of the sequential fabrication process for the BAK1/Cu/Al/BaTiO_3_/FASnI_3_/BP SPR refractive-index sensor.

The proposed multilayer stack is compatible with established thin-film deposition and transfer processes, and a feasible fabrication route is schematically illustrated in Figure 8.

The fabrication begins with a BAK1 prism that is ultrasonically cleaned sequentially in methanol, acetone, and deionized water and then blown dry under a nitrogen stream. A Cu film is deposited on the prism base by physical vapor deposition (PVD), followed immediately by an ultrathin Al cap deposited by resistive thermal evaporation. On top of this metal stack, a BaTiO_3_ dielectric layer is then spin-coated onto the metal stack from a sol–gel precursor, with the spin speed and annealing conditions tuned to obtain a uniform film [41].

The FASnI_3_ layer is grown by chemical vapor deposition (CVD) processing. Equimolar amounts of SnI_2_ and CH(NH_2_)_2_I are thoroughly ground and placed in the hot zone of the CVD chamber, while the BaTiO_3_-coated prism is positioned 20 cm away in the cooler zone. After the chamber is evacuated to 100 Pa and purged three times with high-purity Ar to remove residual oxygen and moisture, the temperature is ramped to 600 °C over 30 min and held for 60 min under an Ar carrier-gas flow of 60 sccm [42]. Finally, a BP flake is prepared by mechanical exfoliation from a bulk black-phosphorus crystal using standard adhesive tape, and a monolayer region is optically identified and transferred onto the FASnI_3_ surface under a microscope so that it uniformly covers the sensing area.

## 4. Conclusions

This work reports a Cu/Al bimetallic SPR refractive-index sensor architecture that integrates BaTiO_3_, FASnI_3_, and BP overlayers to enhance evanescent-field confinement and interfacial RI response. The optimized BAK1/Cu/Al/BaTiO_3_/FASnI_3_/BP heterostructure achieves a peak angular sensitivity of 510.11°/RIU at an analyte RI of 1.355 and a composite sensitivity figure of 76.39 RIU^−1^, representing a 6.17-fold improvement in angular sensitivity and up to a 28.1-fold improvement in CSF over the conventional F2/Au, SF2/Au, BAF10/Au, and SF5/Au benchmarks. Comparative prism analysis identifies BAK1 as the optimal coupling medium among the evaluated substrates, while stepwise characterization confirms cumulative sensitivity enhancement from each functional layer. Fabrication tolerance to ±7.5% thickness deviations further supports the theoretical robustness of the design. This GA-driven multilayer design paradigm offers a generalizable strategy for plasmonic refractive-index sensor development, with potential applications in chemical analysis, environmental monitoring, food-quality assessment, and biochemical analysis. Future work should focus on experimental validation of the film stack, assessment of the aqueous stability of the FASnI_3_ and BP layers, and evaluation of the sensor response in complex sample matrices.

## Figures and Tables

**Table 1:** Refractive indices of the constituent materials at 633 nm.

Material	Function Layers	Refractive Index (633 nm)	References
BAK1 prism	Coupling prism	1.570	[24]
F2 prism	Comparison prism	1.620	[24]
SF2 prism	Comparison prism	1.648	[25]
BAF10 prism	Comparison prism	1.667	[24]
SF5 prism	Comparison prism	1.668	[24]
Cu	Primary plasmonic layer	0.0369 + 4.5393i	[26]
Al	Metal capping layer	0.0778 + 5.8535i	[24]
BaTiO_3_	Dielectric enhancement layer	2.4042	[24]
FASnI_3_	Dielectric enhancement layer	2.782	[26]
BP	Top sensing layer	3.5 + 0.01i	[26]

**Table 2:** Ranges of the design parameters.

Design Parameters	Lower Bound	Upper Bound
h_1_ (Cu)	10 nm	70 nm
h_2_ (Al)	1 nm	20 nm
h_3_ (BaTiO_3_)	1 nm	20 nm
h_4_ (FASnI_3_)	10 nm	60 nm
L_BP_	1	10

**Table 3:** Parameters of GA.

Parameter	Value
Population size	400
Crossover	0.8
Mutation rate	0.05
Maximum number of generations	200

**Table 4:** Performance metrics of the GA-optimized BAK1/Cu/Al/BaTiO_3_/FASnI_3_/BP sensor across the analyte refractive index range of 1.330–1.3575.

Refractive Index	S (°/RIU)	FWHM (°)	R_min_	DRD	CSF (RIU^−1^)
1.33	235.44	1.96	0.1179	0.8821	105.85
1.3325	235.44	2.036	0.125	0.875	101.20
1.335	255.06	2.13	0.1336	0.8664	103.57
1.3375	264.86	2.23	0.1442	0.8558	101.57
1.34	274.67	2.33	0.1571	0.8429	99.38
1.3425	313.91	2.48	0.1732	0.8268	104.78
1.345	323.72	2.65	0.1939	0.8061	98.52
1.3475	372.77	2.87	0.2212	0.7788	101.18
1.35	412.01	3.14	0.2585	0.7415	97.32
1.3525	480.68	3.51	0.3126	0.6874	94.21
1.355	510.11	4.02	0.3977	0.6023	76.39
1.3575	245.25	4.59	0.5447	0.4553	24.35

**Table 5:** Comparison of sensitivity and CSF values between the proposed SPR sensor and recently reported structures.

References	Reported Year	Structure	Sensitivity (°/RIU)	CSF (RIU^−1^)
Kushwaha et al. [28]	2018	SF10/ZnO/Au/MoS_2_/graphene/SM	101.58	-
Mudgal et al. [29]	2020	BK7/Au/MoS2/h-BN/Graphene/SM	194.12	16.04
Kumar et al. [30]	2020	BK7/ZnO/Ag/BaTiO_3_/WS_2_	235.00	-
Kumar et al. [31]	2021	BK7/ZnO/Si/MXene/Sensing layer	231.00	-
Rikta et al. [32]	2021	SF10/Au/Ag/Cu/SnSe/BP	96.43	-
Panda and Pukhrambam [33]	2022	CaF_2_/TiO_2_/Ag/PtSe_2_/WS_2_	240.00	-
Kumar et al. [34]	2022	Ti/Ag/graphene/MXene/MoS_2_ /SM	144.72	-
Karki et al. [35]	2024	BK7/Ag/MXene/ZnO/ Graphene/SM	184.00	22.64
Taya et al. [36]	2023	BK7/Ag/BiFeO/graphene	293.00	-
Bouandas et al. [26]	2024	BK7/Cu/FASnI_3_/BP	459.22	-
Khodaei and Heidarzadeh [37]	2025	BK7/Au/graphene/Al_2_O_3_/MXene/SM (M = 1, G = 1)	162.32	-
Tiandho et al. [38]	2025	CaF2/Ag/Graphene/ DNA/SM	350.00	35.94
Yadav and Tripathy [39]	2025	SF10/Au/CsScCl_3_/Graphene	98.39	8.15
Yadav and Tripathy [39]	2025	SF10/Au/CsScBr_3_/Graphene	100.43	7.89
Yadav and Tripathy [39]	2025	SF10/Au/CsScI_3_/Graphene	100.43	7.93
Mohadesi [40]	2025	CsF/Cu/FASnI_3_/BP	>500	-
Proposed	2026	BAK1/Cu/Al/BaTiO_3_/FASnI_3_/BP	510.11	76.39

## Data Availability

The original contributions presented in this study are included in this article. Further inquiries can be directed to the corresponding authors.

## Abbreviations

The following abbreviations are used in this manuscript:

BP	black phosphorus
CSF	composite sensitivity figure
DRD	dip reflection depth
FWHM	full width at half maximum
GA	genetic algorithm
RI	refractive index
SPR	surface plasmon resonance
TMM	transfer matrix method
